# Fatal Renal Abscess Caused by *Porphyromonas gingivalis* and Subcapsular Hemorrhage, Japan

**DOI:** 10.3201/eid3010.240078

**Published:** 2024-10

**Authors:** Yuichiro Atagi, Yoshito Homma, Sadamu Yamashi, Ken Kikuchi, Yoji Nagashima

**Affiliations:** Ehime Prefectural Central Hospital, Matsuyama, Japan (Y. Atagi, Y. Homma, S. Yamashi);; Tokyo Women’s Medical University School of Medicine, Tokyo, Japan (K. Kikuchi, Y. Nagashima)

**Keywords:** *Porphyromonas gingivalis*, bacteria, renal abscess, spontaneous perirenal hemorrhage, Japan

## Abstract

A 61-year-old man in Japan with abdominal pain was suspected of having a renal tumor. Despite initial treatment, his condition rapidly deteriorated, leading to death. Postmortem examination revealed a renal abscess and sepsis caused by *Porphyromonas gingivalis*. This case underscores the need to consider atypical pathogens in renal masses.

Renal abscesses are rare and often difficult to distinguish from malignant renal tumors. Renal abscesses typically are caused by gram-negative bacteria, such as *Escherichia coli* and *Proteus* species, as well as gram-positive *Staphylococcus aureus* (*1*). *Porphyromonas gingivalis*, an anaerobic, gram-negative bacterium primarily associated with periodontal disease, is an uncommon cause of systemic infections (*2*). We report a fatal case of renal abscess and sepsis caused by *P. gingivalis* in a man in Japan.

The patient was a 61-year-old man with a body mass index of 22.3 kg/m^2^ who had a history of hypertension, hyperuricemia, dyslipidemia, and cerebral hemorrhage. However, he had no residual effects from the cerebral hemorrhage and worked without any problems. He was undergoing follow-up for an intraductal papillary mucinous tumor of the pancreatic duct in the internal medicine department at Ehime Prefectural Central Hospital in Matsuyama, Japan. One week before admission, he experienced a brief fever and gum pain. Three days before admission, routine imaging revealed a mass in his right kidney (Figure 1, panel A), leading to a referral to the urology department. 

**Figure 1:**
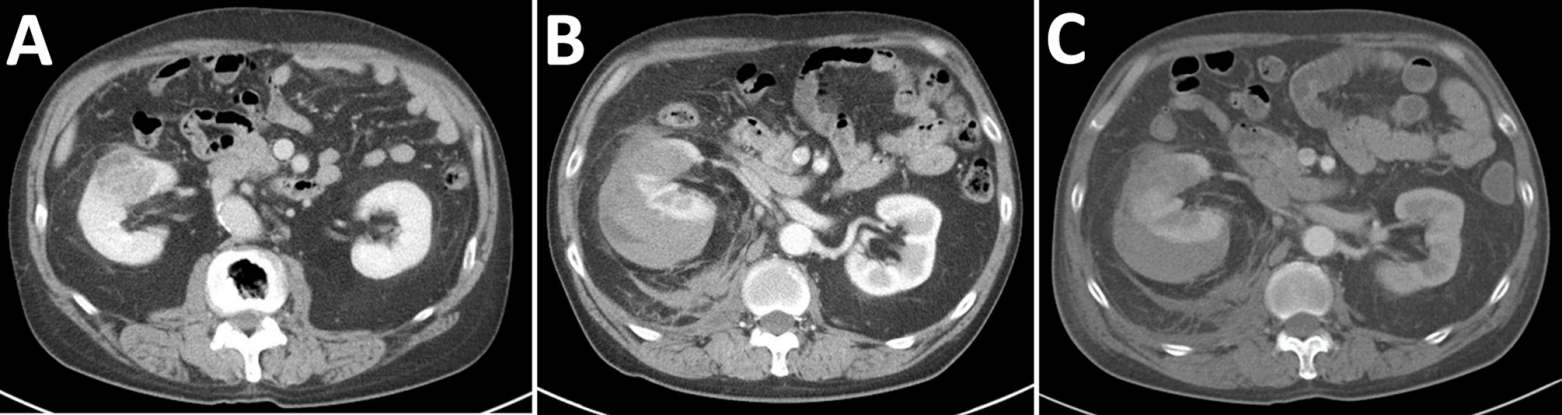
CT scans in a case of fatal renal abscess caused by *Porphyromonas gingivalis* and subcapsular hemorrhage, Japan. A) Contrast enhanced CT 3 days before patient admission shows a 3.5-cm large right renal mass, which was suspected to be renal carcinoma based on imaging findings. B) Contrast enhanced CT at admission. The patient was hospitalized for bleeding from the renal mass. C) Contrast enhanced CT 1 day after admission, showing a shrinking hematoma. CT, computed tomography.

At admission, the patient was in severe pain. A contrast-enhanced computed tomography (CT) scan of the abdomen revealed a subrenal capsular hematoma caused by tumor rupture (Figure 1, panel B). Spontaneous rupture of a renal tumor was diagnosed and considered a grade 1 renal injury. After examination, we admitted the patient for conservative therapy. We performed a follow-up contrast-enhanced CT scan of the abdomen a day after admission, which showed no changes in hematoma size or effusion progression (Figure 1, panel C). We continued conservative treatment, but 2 days after admission, the patient showed signs of poor oxygenation, tachycardia, and hypotension. On day 3 of admission, the patient’s respiratory function deteriorated, and he required intubation. 

During the patient’s hospitalization, no fever was observed. However, blood tests indicated an elevated inflammatory response. We suspected a hematoma infection, drew blood for cultures, and started the patient on meropenem. However, the patient’s general condition did not improve, and he died on the fourth day after admission. 

Two sets of blood cultures obtained before initiating antimicrobial drug therapy were both negative. A urine culture detected only the presence of streptococci. The family requested an autopsy to determine the cause of death. 

The autopsy was approved and revealed that the right renal mass was not a tumor but a renal abscess. We found turbid ascites and pleural effusions in the abdominal and thoracic cavities. The pathology report revealed that the cause of death was renal abscess, associated sepsis, and respiratory failure due to acute respiratory distress syndrome. The renal subcapsular hematoma caused disruption of cortical vessels around the abscess. Because blood culture results were negative, we performed immunostaining of the pathology specimen from the renal abscess (Figure 2). We also performed 16srRNA gene sequencing of isolates from renal abscess specimens, which identified the causative organism as *P. gingivalis*.

**Figure 2:**
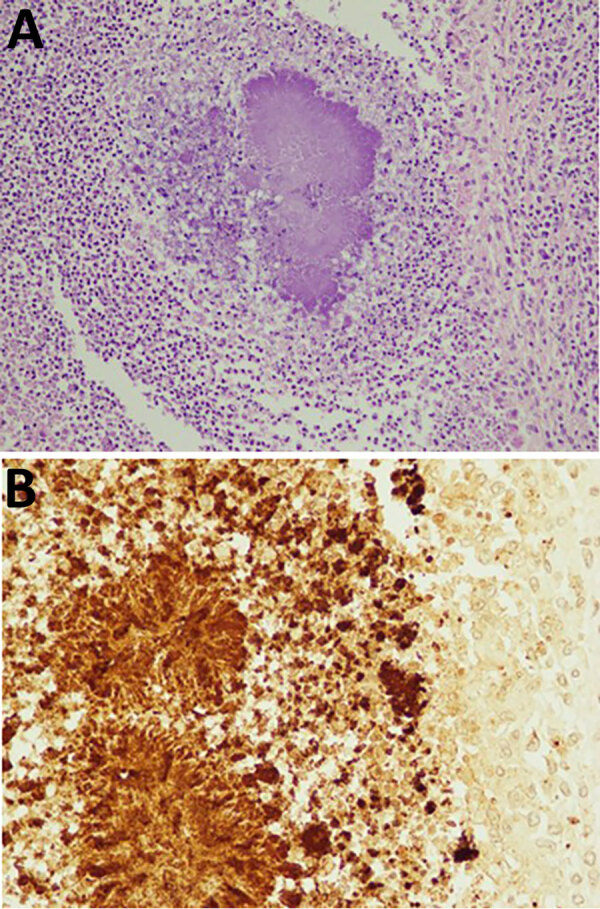
Immunology of mass in a case of fatal renal abscess caused by *Porphyromonas gingivalis* and subcapsular hemorrhage, Japan. A) Hematoxylin and eosin stain showing gram-negative bacteria in center. Original magnification ×100. B) Immunohistochemistry with antibody diluted 100 times. Further magnification showing gram-negative bacteria. Original magnification ×200.

*P. gingivalis* is a known etiologic agent of periodontitis and has been observed to cause abscesses in various body parts, including the brain and liver (*3*–*5*). However, reports of renal abscesses caused by this pathogen are lacking. Although primarily associated with periodontitis, *P. gingivalis* has also been implicated in various systemic diseases and systemic infections, highlighting its potential as a versatile pathogen (*6*). The pathogenesis of *P. gingivalis* involves several virulence factors, including fimbriae, hemagglutinins, and gingipains, which enable the bacterium to invade tissues and evade the host immune response (*7*,*8*). *P. gingivalis* also is reported to have biofilm formation, intravenous dipeptidyl peptidase activity, strong induction of inflammatory cytokine secretion, and the ability to infiltrate epithelial cells to evade the immune response activation (*9*). The route of infection in this case remains unclear, but hematogenous spread from a subclinical oral infection is plausible.

Renal abscesses are typically associated with underlying conditions, such as diabetes mellitus, urinary tract obstructions, or immunosuppression (*10*). However, our patient had no notable immunodeficiency or recent urologic interventions that could predispose him to such an infection. The atypical clinical manifestations, without classic signs of sepsis-like fever or leukocytosis, likely delayed the diagnosis and appropriate treatment.

Early recognition and aggressive treatment are critical in managing renal abscesses. Appropriate imaging studies, prompt abscess drainage, and targeted antimicrobial therapy are key to successful renal abscess outcomes. Identification of *P. gingivalis* in this case highlights the importance of comprehensive diagnostic workups, including advanced molecular techniques, to detect uncommon pathogens.

In summary, we identified a rare case of fatal renal abscess and sepsis caused by *P. gingivalis*. Early diagnosis and aggressive management are crucial to improving patient outcomes in such complex infections. This case underscores the need for clinicians to maintain a high index of suspicion for atypical pathogens in patients with renal masses and systemic symptoms, even when classic risk factors are absent.
